# GAIT SPEED AS A FUNCTIONAL VITAL SIGN: FROM IMPLEMENTATION TO INTERVENTION

**DOI:** 10.1093/geroni/igae098.1912

**Published:** 2024-12-31

**Authors:** Emma Baillargeon, Alexander Garbin, Jonathan Bean

**Affiliations:** Chair; Co-Chair; Discussant

## Abstract

Older adults with impaired mobility have a greater risk of falls, hospitalization, loss of independence, and mortality. Interventions to improve mobility are effective, but because mobility limitations are under-diagnosed, patients are often not referred for treatment unless they fall or report a change in function. Walking (gait) speed is a strong predictor of overall health (a “functional vital sign”) and can identify patients who may benefit from intervention, but in most clinics is not routinely assessed like other vital signs. In this symposium, we will present four projects on using routine gait speed assessment to identify and treat mobility limitations in outpatient clinical care. The first two speakers (Baillargeon and Garbin) will present projects implementing gait speed in outpatient geriatric clinics, the factors associated with implementation, project outcomes, and strategies to integrate gait assessment into clinical workflows. The third speaker (Lo) will present on self-administered app-based walking assessments that can allow for more frequent measures of walking in various settings and how this data may inform clinical decision making. Finally, the last speaker (Brach) will discuss interventions to improve gait speed that could be used to treat mobility limitations once patients are identified. Our discussant (Bean) will lead a conversation about this work in the larger context and possible future directions for the field. Together, the talks in this symposium will provide evidence that routine, quantitative gait assessment in outpatient care is feasible and has significant potential to improve the care for older adult patients across the continuum of healthcare.

